# Smartphone-recorded physical activity for estimating cardiorespiratory fitness

**DOI:** 10.1038/s41598-021-94164-x

**Published:** 2021-07-21

**Authors:** Micah T. Eades, Athanasios Tsanas, Stephen P. Juraschek, Daniel B. Kramer, Ernest Gervino, Kenneth J. Mukamal

**Affiliations:** 1 Primary Care, VA Boston Healthcare System, 1400 VFW Pkwy, West Roxbury, Boston, MA 02132 USA; 2 Usher Institute, Edinburgh Medical School, University of Edinburgh, Edinburgh, UK; 3 Richard A. and Susan F. Smith Center for Outcomes Research in Cardiology, Beth Israel Deaconess Medical Center, Boston, MA USA

**Keywords:** Cardiovascular diseases, Diagnostic markers, Preventive medicine

## Abstract

While cardiorespiratory fitness is strongly associated with mortality and diverse outcomes, routine measurement is limited. We used smartphone-derived physical activity data to estimate fitness among 50 older adults. We recruited iPhone owners undergoing cardiac stress testing and collected recent iPhone physical activity data. Cardiorespiratory fitness was measured as peak metabolic equivalents of task (METs) achieved on cardiac stress test. We then estimated peak METs using multivariable regression models incorporating iPhone physical activity data, and validated with bootstrapping. Individual smartphone variables most significantly correlated with peak METs (*p*-values both < 0.001) included daily peak gait speed averaged over the preceding 30 days (r = 0.63) and root mean square of the successive differences of daily distance averaged over 365 days (r = 0.57). The best-performing multivariable regression model included the latter variable, as well as age and body mass index. This model explained 68% of variability in observed METs (95% CI 46%, 81%), and estimated peak METs with a bootstrapped mean absolute error of 1.28 METs (95% CI 0.98, 1.60). Our model using smartphone physical activity estimated cardiorespiratory fitness with high performance. Our results suggest larger, independent samples might yield estimates accurate and precise for risk stratification and disease prognostication.

## Introduction

Cardiorespiratory fitness is strongly and independently associated with mortality, cardiovascular mortality, and cancer-related mortality^1–7^. It is also implicated in the development, prognosis, and treatment of many medical conditions such as diabetes^8–11^, cancer^12–16^, obesity^17,18^, anxiety^19^, depression^20^, and falls^21–23^. The gold standard of fitness measurement is VO_2_max, an individual’s maximum rate of oxygen uptake, which defines functional aerobic capacity. VO_2_max is often expressed in metabolic equivalents of task (METs), which are multiples of normal baseline oxygen uptake at rest. VO_2_max or METs are classically measured in a laboratory by functional exercise testing, such as with a treadmill, where oxygen uptake is measured as the workload is incrementally increased^2,24^. Because the time commitment and need for specialized equipment limit accessibility of formal exercise testing, predictive models have been developed. Such models may input anthropometric data (i.e. age, sex, and body-mass index) and day-to-day physical activity, which itself is associated with physical fitness. Measurements of physical activity have included questionnaires^25–30^, walking tests^31–34^, accelerometry^35–37^, and fitness tracking devices^38–40^. The advent of smartphones has introduced near ubiquitous physical activity measurement as most smartphones integrate accelerometers and gyroscopes which can track owner motion. Indeed, an increasing number of apps claim to measure fitness in terms of VO_2_max, yet few have been reliably validated^41^.

Accurate estimates of cardiorespiratory fitness available at the point of care have enormous potential value. Estimated fitness could be used to estimate mortality and guide end-of-life planning. Smartphone estimated fitness could also guide decisions for risky medical interventions such as chemotherapy and surgery. Individuals with low fitness and high baseline mortality may be less inclined to take on additional risk. Fitness estimates can also be used to identify those at risk for diabetes, obesity, and falls to target with preventive measures such as exercise programs, nutrition programs, and fall prevention. They could also be used to track functional status such as with obese patients in an exercise program, or cancer patients receiving chemotherapy. In short, widely available point of care estimates of fitness have potential value across multiple domains of health care. In this study, we used smartphone-derived physical activity observations to estimate fitness as measured by exercise testing among older adults in a health care setting.

## Materials and methods

Patients undergoing treadmill testing in the Beth Israel Deaconess Cardiovascular Stress Testing Laboratory between September 2017 and June 2018 were recruited in-person at the laboratory or by telephone after leaving the laboratory. As such, participants included individuals undergoing diagnostic workup or risk stratification for coronary heart disease or other heart diseases. We restricted to owners of iPhone models 5S and above or Apple watches because we observed these models to automatically and continuously track physical activity within the Apple Health application (Apple Inc., Cupertino, CA). This allowed for retrospective data collection. Participants were included if they provided consent and were excluded if they did not complete a stress test. Physical activity data exported from the Apple Health application is formatted per episode of activity. Each episode of activity is an observation with a recorded start time, stop time, number of steps, and distance in miles. These parameters are all measured using Apple’s proprietary algorithms with input from onboard sensors such as accelerometers, gyroscopes, barometers, and GPS. Activity episodes are recorded with a duration between 1 and 600 s, and a new observation is created if the activity extends beyond 600 s. When cleaning data we also excluded participants whose data was recorded by a non-Apple device, and those missing data two weeks immediately preceding the stress test (Supplementary Fig. S1). The Apple Health application has an export function, and we used this to securely e-mail physical activity data to study servers which were owned and maintained by the Beth Israel Deaconess Medical Center, Boston, Massachusetts.

Informed consent was obtained from all subjects. The study protocol was approved by the Institutional Review Board at Beth Israel Deaconess Medical Center in accordance with relevant guidelines and regulations.

Age, self-reported race and sex, height, weight, and resting blood pressure and heart rate were collected from the electronic medical record^42–45^. Blood pressure and heart rate were measured at rest before the stress test using an automated oscillometer. From the downloaded physical activity data, we used the time, steps, and distance for each activity episode to calculate velocity in both steps/second (i.e., cadence) and meters/second, observed active time, and stride. Then, using pre-specified intervals prior to treadmill testing (1, 7, 30, 90, 180, and 365 days), we computed the sum of observed active time, sum of steps, sum of distance, peak velocity in steps/second and meters/second, and average stride length. RMSSD, a measure of variability, was calculated for each of these measures respectively by taking the daily sum, peak, or average, finding the difference between successive days, squaring the differences, and then taking the square root of the average. For those with fewer than 365 days of observation, we imputed missing data by carrying existing observations backward in time. In some activity episodes, steps were missing, but distance was present or vice versa. We imputed missing steps or distance by using whichever variable was available and the mean stride length of that individual for that day. Observations exceeding maximal physiologic ranges, such as velocity ≥ 10.44 m/second or stride length ≥ 2.47 m, were excluded^46^.

Cardiorespiratory fitness as peak metabolic equivalents of task (METs) was estimated by maximal treadmill stress testing using the extensively validated Bruce protocol^47^ or a modified protocol. Modified protocols with lower intensity stages at the beginning were selected for frail or deconditioned individuals based upon a pre-test query of daily activities to allow for titration of heart rate over approximately 10 min.

Next, taking the above anthropometric and computed physical activity variables, we calculated univariable Pearson correlations with peak METs and excluded those with a *p*-value > 0.05. Then we computed covariance between remaining variables. For each remaining variable we gathered other variables with covariance > 0.7 and selected the one with the highest univariable Pearson correlation with peak METs. This variable was included in a pool of candidates for the multivariable regression model. Using the pool of candidate variables, we built a multivariable regression model to estimate peak METs using bidirectional stepwise selection maximizing adjusted R-squared. We validated model performance (as estimated by MAE) using bootstrapping with 10,000 samples and performed sensitivity analysis with tenfold cross-validation. Statistical analysis was performed using RStudio version 1.3.1093 (RStudio, Boston, MA).

## Results

### Baseline characteristics

Baseline characteristics of the study population are displayed in Table 1. Of 50 participants, median age was 67 (inter-quartile limits 55, 71) years and 19 (38%) were female. Data cleaning yielded 1.1 million unique activity episodes.

**Table 1:** Baseline characteristics of study population (*N* = 50).

Activity Episode Observations	1,072,510
Epochs/Participant	17,096 (5,570, 29,859)
Days/Participant (days)*	535 (278, 822)
Daily Observation Time/Participant (hours)	2.7 (1.9, 3.7)
Female	38%
Age (years)	67 (55, 71)
Height (centimeters)	168 (163, 173)
Weight (kilograms)	76.2 (68.0, 84.8)
Resting Heart Rate (beats per minute)	68 (60, 74)
Resting Systolic Blood Pressure (mmHg)	123 (114, 138)
Resting Diastolic Blood Pressure (mmHg)	78 (70, 80)
*Device Version*	
iPhone 5 s	10%
iPhone 6	20%
iPhone 6 Plus	8%
iPhone 6 s	20%
iPhone 6 s Plus	10%
iPhone SE	4%
iPhone 7	18%
iPhone 7 Plus	8%
Apple Watch Series 3	2%
Cardiorespiratory Fitness (MET)	10.60 (8.25, 11.90)
Peak Gait Speed at 30 Days (meters/second)	0.76 (0.60, 1.00)
RMSSD Daily Distance at 365 Days (km)	1.89 (1.61, 2.72)
Stride Length at 90 Days (meters)	0.65 (0.62, 0.68)

Baseline characteristics of study population including median (Interquartile range) for continuous variables and percentage for categorical variables.

*Days/Participant Range (13, 1,147).

**Abbreviations—mmHg: millimeters of mercury, SE: special edition, MET: metabolic equivalents of task, RMSSD: root mean square of the successive differences, km: kilometers.

### Univariable analysis

In univariable analysis, smartphone variables most significantly correlated with peak METs (*p*-values all < 0.001; covariance < 0.7) included daily peak gait speed in meters/second averaged over 30 days (r = 0.63), RMSSD of daily distance in miles averaged over 365 days (r = 0.57), and stride length averaged over 90 days (r = 0.43). Supplemental Fig. S2 shows the relationship of these variables with peak METs averaged over pre-specified intervals from 1 to 365 days. In general, peak gait speeds and stride length measured closer to stress test date seemed to correlate more closely with peak METs, while the relationship of RMSSD of daily distance with peak METs was stronger with more observation time. Supplementary Table S1 lists Pearson correlations and *p*-values for all variables with *p*-value < 0.05.

### Multivariable analysis

**Figure 1:**
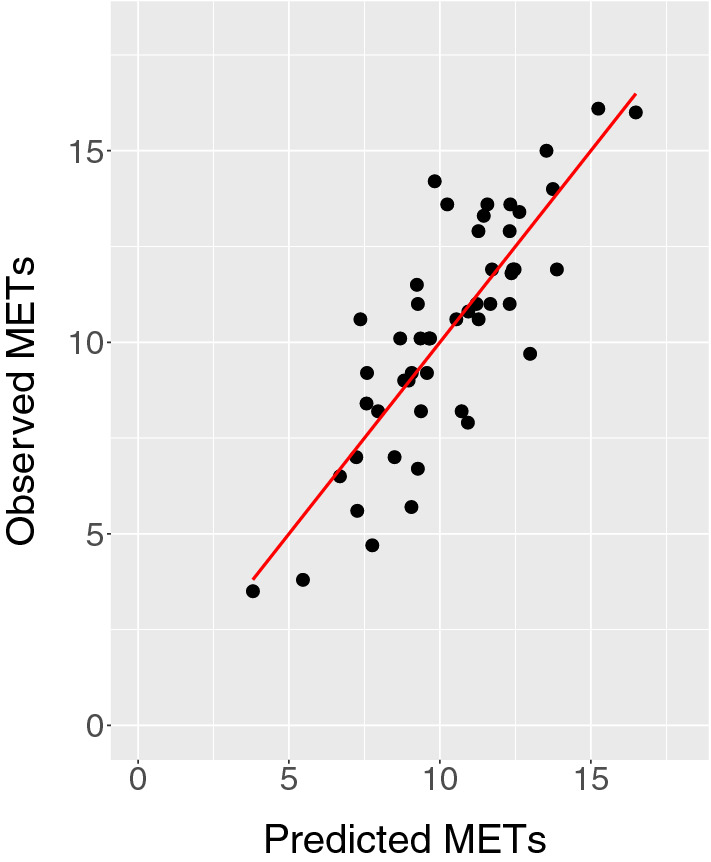
Observed Versus Predicted METs in Entire Study Population. The plot depicts observed versus predicted METs in the entire study population. The red line represents the line of unity.

Figure 1 displays the best-performing multivariable regression model. This model explained 68% of variability in observed METs (95% confidence limits 46%, 81%), and included age, body mass index, and RMSSD of daily distance in miles averaged over 365 days (equation parameters reported in Supplemental Table S2 and Bland–Altman plot included in Supplemental Fig. S3). Bootstrapping indicated strong performance, with a MAE of 1.28 METS (95% confidence limits 0.98, 1.60). Model performance was comparable for females (MAE = 1.29 METs) and males (MAE = 1.27METs) and was not appreciably larger in tenfold cross-validation (1.35 METs).

## Discussion

A model using smartphone data estimated cardiorespiratory fitness with high performance in a health care setting. Our study is significant in that it demonstrates the feasibility of estimating cardiorespiratory fitness using smartphones. Such cardiorespiratory fitness predictions have potential utility in estimating mortality, identifying those at risk for diseases, estimating prognosis of diseases, and tracking the progress of interventions.

Previous attempts to estimate fitness using self-reported physical activity achieved varying degrees of success. These estimates have relied upon self-reported questionnaire data subject to recall bias^25–30^, time-consuming walking tests^31–34^, or specialized accelerometry or fitness tracking equipment^35–40^. In contrast, we present an objective, point-of-care fitness estimation relying upon nearly ubiquitous smartphone with exceptional performance. Utilizing smartphone physical activity data has multiple advantages: (1) large numbers of measurements are collected passively, permitting retrospective averaging that strengthens associations (2) these data are at least as accessible as basic clinic variables (e.g. height, weight, and blood pressure), (3) the data are widely available (85% of US adults own a smartphone, and physical activity trackers are easily activated on Android devices)^48^, and (4) they appear to perform well when compared to a gold-standard clinical measurement and compared to previous estimates. Based upon day-to-day physical activity, Kwon et al. built a model predicting cardiorespiratory fitness with an R^2^ of 0.66 using activity data from Fitbit activity trackers^38^. Bonomi et al. attained an R^2^ of approximately 0.77 using both a heart rate monitor and activity tracker^37^. However, all these models relied upon specialized fitness tracking equipment and minute-to-minute heart rate measurements. In contrast, we achieved an R^2^ of 0.68 using smartphone physical activity data. Interestingly, Altini et al. used the HRV4Training smartphone app, in addition to a heart rate monitor, to predict VO_2_max, but achieved only an R^2^ of 0.64^49^.

Our observation that the variability of daily distance was most predictive of fitness was surprising, as we had expected that peak gait speed would be the best predictor. In fact, the variability of daily distance was not only strongly correlated with peak gait speed but also superior in predictive power when combined with age and body mass index. We hypothesize the fitness-predicting value of the variability of daily distance lies in its ability to capture an individual’s reserve capacity to increase walking distance upon demand. Those with low fitness may be unable to drastically vary their travelling distance, whereas more fit individuals exercise a flexible option to travel further when needed.

Some have questioned the accuracy of fitness trackers, especially smartphones, in measuring step data^50–52^. At least one study has demonstrated modest accuracy of step counting using iPhone fitness data^53^. The same study indicated dedicated fitness tracking devices, such as the Fitbit activity tracker, may be more accurate than smartphones for counting steps. In this regard, our distinctive focus on fitness, rather than on simply estimating activity, is crucial, as a smartphone need not be carried at all times. For some variables, such as peak gait speed, data collection closer to stress test date seemed to correlate more closely with peak METs. For other variables, such as RMSSD of daily distance, association with peak METs seemed stronger with more observation time, suggesting that averaging more data may reduce smartphone measurement error. It is also possible that the accuracy of smartphone data is dependent upon phone-carrying location (e.g. hip versus purse), yet our data show powerful estimation of fitness even without knowing phone-carrying location and in both sexes.

Our study is limited by a small sample of patients at risk for heart disease and restriction to a single, albeit well-known, manufacturer (Apple Inc.). Also, since physical activity was assessed exclusively through an iPhone, our study was unable to include physical activity when not carrying an iPhone. Furthermore, we used a stress treadmill estimation of fitness (METs) rather than VO_2_max. However, cardiopulmonary exercise treadmill test measurement of VO_2_max is rarely performed. Nonetheless, these promising results demonstrate that the incremental predictive utility of smartphone data, combined with its ready accessibility, open new exciting opportunities for clinical and research estimation of cardiorespiratory fitness. Based upon the feasibility demonstrated in this study, it seems likely that larger, independent studies can improve sufficiently on our algorithm to yield truly useful estimates of fitness for clinical and epidemiological purposes.

## Supplementary Information

Supplementary Information.

## Data Availability

Deidentified datasets generated during and/or analyzed during the current study are available from the corresponding author with completion of an approved data distribution agreement from the sponsoring institution.
