# Supplementary material for: Smartphone-recorded physical activity for estimating cardiorespiratory fitness
Source: Sci Rep. 2021 Jul 21;11:14851. doi: 10.1038/s41598-021-94164-x (PMC8295266; doi:10.1038/s41598-021-94164-x)
Supplement: Supplementary file 1 — Supplementary Information. [file 41598_2021_94164_MOESM1_ESM.docx]

**Supplemental Information**

**Title:**

Smartphone-Recorded Physical Activity for Estimating Cardiorespiratory Fitness

**Authors:**

Micah T. Eades, MD, MS*^1^, Athanasios Tsanas, PhD^2^, Stephen P. Juraschek, PhD, MD^3^, Daniel B. Kramer, MD, MPH^3^, Ernest Gervino, ScD^3^, Kenneth J. Mukamal MD, MPH^3^

**Supplementary Figure S1. Diagram of Excluded Participants**

55 Provided consent

50 Study participants

1 Stress test incomplete

3 Exported data from non-Apple devices*

1 Stopped collecting data 123 days prior to stress test

*Two individuals used a Misfit, and one used a Garmin fitness tracker. These devices erased iPhone data, replacing it with their own.

**Supplementary Figure S2. Correlations of METs with Peak Gait Speed, Stride Length, and RMSSD of Distance by Days Preceding Stress Test**

**
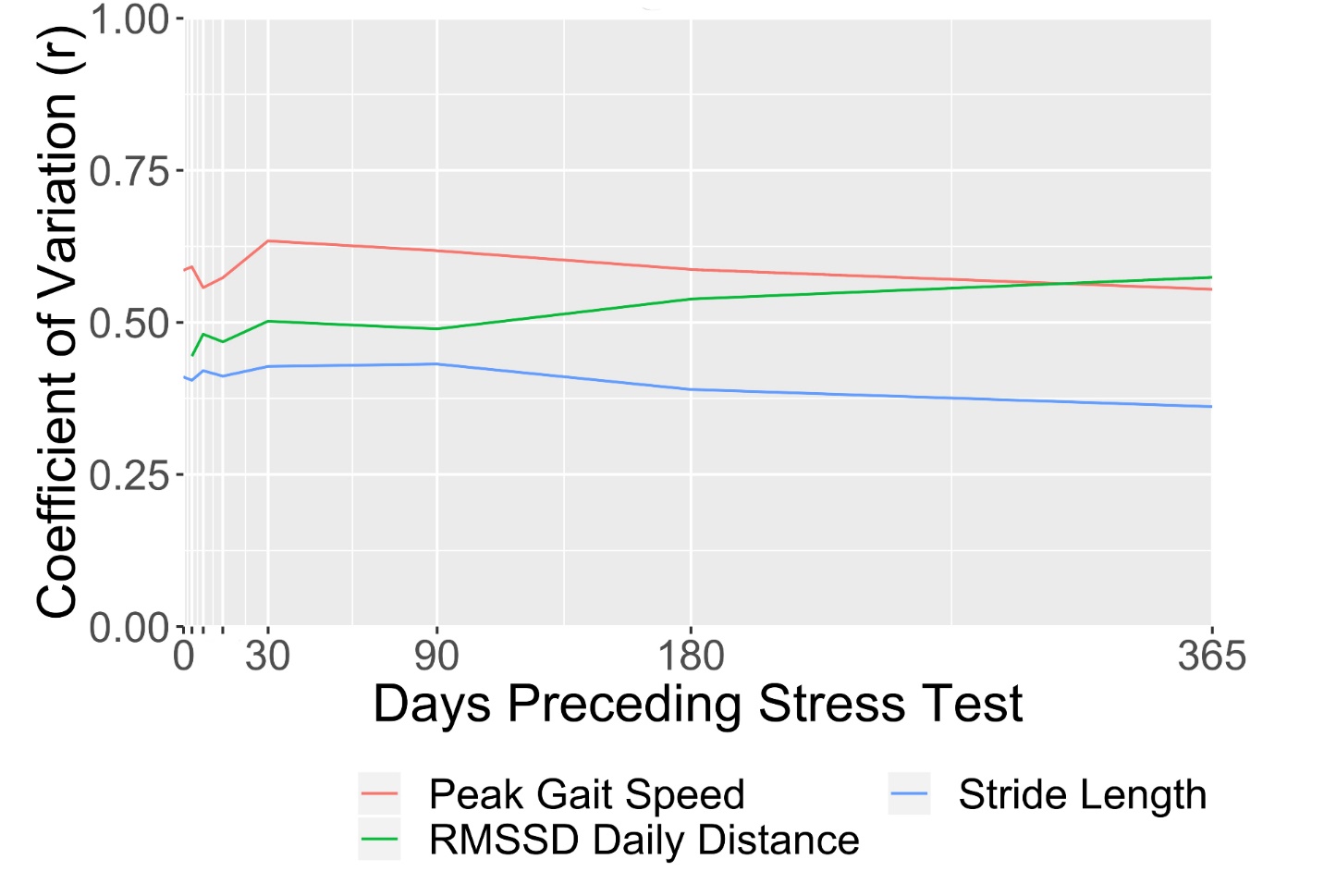
**

**Supplementary Figure S3. Bland Altman Plot Comparing Observed and Predicted METs**

**
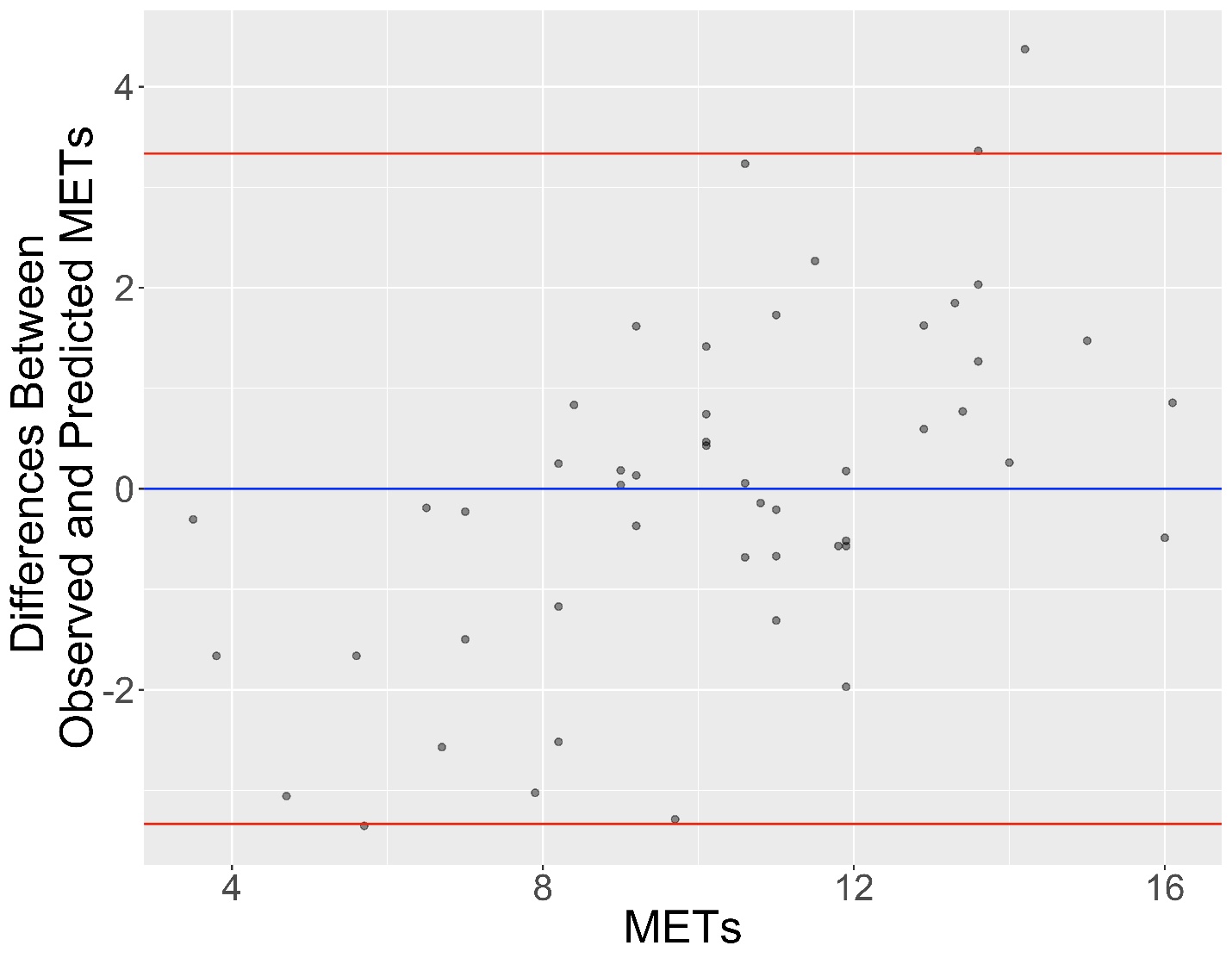
**

**Blue line demonstrates mean of differences between observed and predicted METs. Red lines represent 1.96*standard deviation of differences between observed and predicted METs.**

**Supplementary Table S1. Univariable Pearson Correlation of Candidate Variables with METs**

| **Variable** | **Univariable Pearson Correlation with METs** | **p-value** |
| --- | --- | --- |
| Age | -0.591 | <0.001 |
| Weight | -0.421 | 0.002 |
| BMI | -0.542 | <0.001 |
| Resting Systolic Blood Pressure | -0.368 | 0.009 |
| Avg Daily Steps at 1 day | 0.378 | 0.007 |
| Avg Daily Steps at 3 days | 0.413 | 0.003 |
| Avg Daily Steps at 7 days | 0.407 | 0.003 |
| Avg Daily Steps at 14 days | 0.421 | 0.002 |
| Avg Daily Steps at 30 days | 0.411 | 0.003 |
| Avg Daily Steps at 90 days | 0.510 | <0.001 |
| Avg Daily Steps at 180 days | 0.507 | <0.001 |
| Avg Daily Steps at 365 days | 0.502 | <0.001 |
| Avg Daily Distance at 1 day | 0.437 | 0.002 |
| Avg Daily Distance at 3 days | 0.464 | <0.001 |
| Avg Daily Distance at 7 days | 0.460 | <0.001 |
| Avg Daily Distance at 14 days | 0.467 | <0.001 |
| Avg Daily Distance at 30 days | 0.454 | <0.001 |
| Avg Daily Distance at 90 days | 0.566 | <0.001 |
| Avg Daily Distance at 180 days | 0.571 | <0.001 |
| Avg Daily Distance at 365 days | 0.564 | <0.001 |
| Peak Step Speed at 1 day | 0.489 | <0.001 |
| Peak Step Speed at 3 days | 0.360 | 0.010 |
| Peak Step Speed at 7 days | 0.398 | 0.004 |
| Peak Step Speed at 14 days | 0.387 | 0.006 |
| Peak Step Speed at 30 days | 0.440 | 0.001 |
| Peak Step Speed at 90 days | 0.417 | 0.003 |
| Peak Step Speed at 180 days | 0.374 | 0.007 |
| Peak Step Speed at 365 days | 0.307 | 0.030 |
| Peak Distance Speed at 1 day | 0.580 | <0.001 |
| Peak Distance Speed at 3 days | 0.488 | <0.001 |
| Peak Distance Speed at 7 days | 0.480 | <0.001 |
| Peak Distance Speed at 14 days | 0.453 | <0.001 |
| Peak Distance Speed at 30 days | 0.480 | <0.001 |
| Peak Distance Speed at 90 days | 0.466 | <0.001 |
| Peak Distance Speed at 180 days | 0.441 | 0.001 |
| Peak Distance Speed at 365 days | 0.392 | 0.005 |
| Peak Duration at 3 days | -0.341 | 0.015 |
| Peak Duration Speed at 7 days | -0.343 | 0.015 |
| Peak Duration Speed at 14 days | -0.343 | 0.015 |
| Peak Duration Speed at 30 days | -0.343 | 0.015 |
| Peak Duration Speed at 90 days | -0.343 | 0.015 |
| Peak Duration Speed at 180 days | -0.343 | 0.015 |
| Peak Duration Speed at 365 days | -0.343 | 0.015 |
| Avg Daily Peak Step Speed at 1 day | 0.517 | <0.001 |
| Avg Daily Peak Step Speed at 3 days | 0.544 | <0.001 |
| Avg Daily Peak Step Speed at 7 days | 0.525 | <0.001 |
| Avg Daily Peak Step Speed at 14 days | 0.544 | <0.001 |
| Avg Daily Peak Step Speed at 30 days | 0.602 | <0.001 |
| Avg Daily Peak Step Speed at 90 days | 0.607 | <0.001 |
| Avg Daily Peak Step Speed at 180 days | 0.585 | <0.001 |
| Avg Daily Peak Step Speed at 365 days | 0.549 | <0.001 |
| Avg Daily Peak Distance Speed at 1 day | 0.586 | <0.001 |
| Avg Daily Peak Distance Speed at 3 days | 0.591 | <0.001 |
| Avg Daily Peak Distance Speed at 7 days | 0.557 | <0.001 |
| Avg Daily Peak Distance Speed at 14 days | 0.574 | <0.001 |
| Avg Daily Peak Distance Speed at 30 days | 0.634 | <0.001 |
| Avg Daily Peak Distance Speed at 90 days | 0.618 | <0.001 |
| Avg Daily Peak Distance Speed at 180 days | 0.587 | <0.001 |
| Avg Daily Peak Distance Speed at 365 days | 0.555 | <0.001 |
| Avg Stride Length at 1 day | 0.410 | 0.003 |
| Avg Stride Length at 3 days | 0.405 | 0.004 |
| Avg Stride Length at 7 days | 0.421 | 0.002 |
| Avg Stride Length at 14 days | 0.412 | 0.003 |
| Avg Stride Length at 30 days | 0.428 | 0.002 |
| Avg Stride Length at 90 days | 0.432 | 0.002 |
| Avg Stride Length at 180 days | 0.390 | 0.005 |
| Avg Stride Length at 365 days | 0.362 | 0.010 |
| RMSSD Daily Steps at 3 days | 0.343 | 0.015 |
| RMSSD Daily Steps at 7 days | 0.374 | 0.007 |
| RMSSD Daily Steps at 14 days | 0.401 | 0.004 |
| RMSSD Daily Steps at 30 days | 0.441 | 0.001 |
| RMSSD Daily Steps at 90 days | 0.411 | 0.003 |
| RMSSD Daily Steps at 180 days | 0.463 | <0.001 |
| RMSSD Daily Steps at 365 days | 0.513 | <0.001 |
| RMSSD Daily Distance at 3 days | 0.444 | 0.001 |
| RMSSD Daily Distance at 7 days | 0.481 | <0.001 |
| RMSSD Daily Distance at 14 days | 0.468 | <0.001 |
| RMSSD Daily Distance at 30 days | 0.502 | <0.001 |
| RMSSD Daily Distance at 90 days | 0.489 | <0.001 |
| RMSSD Daily Distance at 180 days | 0.538 | <0.001 |
| RMSSD Daily Distance at 365 days | 0.574 | <0.001 |

**Supplementary Table S2.** **Equation Parameters of Linear Regression Model for Estimating Peak METs**

|  | Coefficient | p-value |
| --- | --- | --- |
| Intercept | 22.204 | <0.001 |
| Age (years) | -0.101 | <0.001 |
| BMI (kg/m^2^) | -0.284 | <0.001 |
| RMSSD daily distance averaged over 365 days (km) | 1.704 | 0.001 |

**Estimated METs = 22.204 – 0.101*X_Age_– 0.284*X_BMI_ + 1.704*X_365dayRMSSDDailyDistance_**
